# Cytotoxicity Effects of Water-Soluble Multi-Walled Carbon Nanotubes Decorated with Quaternized Hyperbranched Poly(ethyleneimine) Derivatives on Autotrophic and Heterotrophic Gram-Negative Bacteria

**DOI:** 10.3390/ph13100293

**Published:** 2020-10-06

**Authors:** Nikolaos S. Heliopoulos, Georgia Kythreoti, Kyriaki Marina Lyra, Katerina N. Panagiotaki, Aggeliki Papavasiliou, Elias Sakellis, Sergios Papageorgiou, Antonios Kouloumpis, Dimitrios Gournis, Fotios K. Katsaros, Kostas Stamatakis, Zili Sideratou

**Affiliations:** 1 Institute of Nanoscience and Nanotechnology, National Centre of Scientific Research ‘‘Demokritos”, 15310 Aghia Paraskevi, Greece; nikosheliopoulos@gmail.com (N.S.H.); geokyth@bio.demokritos.gr (G.K.); kymarin@gmail.com (K.M.L.); knpanagiotaki@gmail.com (K.N.P.); a.papavasiliou@inn.demokritos.gr (A.P.); e.sakellis@inn.demokritos.gr (E.S.); s.papageorgiou@inn.demokritos.gr (S.P.); f.katsaros@inn.demokritos.gr (F.K.K.); 2 Department of Industrial Design & Production Engineering, University of West Attica, 12241 Egaleo, Attiki, Greece; 3 Institute of Biosciences and Applications, National Centre of Scientific Research ‘‘Demokritos”, 15310 Aghia Paraskevi, Greece; kstam@bio.demokritos.gr; 4 Department of Material Science & Engineering, University of Ioannina, 45110 Ioannina, Greece; antoniokoul@gmail.com (A.K.); dgourni@uoi.gr (D.G.)

**Keywords:** carbon nanotubes, quaternary ammonium groups, hyperbranched dendritic polymers, antibacterial properties, anti-cyanobacterial properties

## Abstract

Oxidized multi-walled carbon nanotubes (oxCNTs) were functionalized by a simple non-covalent modification procedure using quaternized hyperbranched poly(ethyleneimine) derivatives (QPEIs), with various quaternization degrees. Structural characterization of these hybrids using a variety of techniques, revealed the successful and homogenous anchoring of QPEIs on the oxCNTs’ surface. Moreover, these hybrids efficiently dispersed in aqueous media, forming dispersions with excellent aqueous stability for over 12 months. Their cytotoxicity effect was investigated on two types of gram(−) bacteria, an autotrophic (cyanobacterium *Synechococcus* sp. PCC 7942) and a heterotrophic (bacterium *Escherichia coli*). An enhanced, dose-dependent antibacterial and anti-cyanobacterial activity against both tested organisms was observed, increasing with the quaternization degree. Remarkably, in the photosynthetic bacteria it was shown that the hybrid materials affect their photosynthetic apparatus by selective inhibition of the Photosystem-I electron transport activity. Cytotoxicity studies on a human prostate carcinoma DU145 cell line and 3T3 mouse fibroblasts revealed that all hybrids exhibit high cytocompatibility in the concentration range, in which they also exhibit both high antibacterial and anti-cyanobacterial activity. Thus, QPEI-functionalized oxCNTs can be very attractive candidates as antibacterial and anti-cyanobacterial agents that can be used for potential applications in the disinfection industry, as well as for the control of harmful cyanobacterial blooms.

## 1. Introduction

Carbon nanotubes (CNTs) have attracted significant scientific and technological interest due to their unique structural characteristics and their excellent electronic, mechanical, and thermal properties [1,2]. Based on these properties, they have been used in a wide range of applications, including fillers in composite materials, sensors, drug delivery systems, antibacterial agents, and others [3,4]. However, their poor dispersibility in solvents, especially in water, has prevented their widespread industrial use, and reduced their great potential. Attempts to overcome this problem have focused on the functionalization of their surface, using a variety of covalent and non-covalent modification strategies [5]. On one hand, various organic molecules, such as dendritic and linear polymers, have been covalently conjugated onto the CNT’s convex surfaces and tips by chemical reactions [6,7,8] in order to reduce aggregates and size polydispersity. However, covalent functionalization causes damage to the conjugated π-electrons, leading to degradation of their properties. On the other hand, non-covalent functionalization, based on π–π stacking and ionic interactions between various molecules and the CNTs graphitic surface [9,10,11] does not affect their electronic structure, and has been achieved using a multitude of surfactants [6,7] and polymers [8,10], resulting in modified CNTs, compatible with specific solvents or targeted applications. In this context, their functionalization with dendritic polymers such as dendrons, dendrimers, and hyperbranched polymers, is expected to be a very promising strategy, when aiming to achieve increased water solubility. This strategy has already been applied to single-walled carbon nanotubes (SWCNTs) that were functionalized using dendritic polymers through non-covalent interactions, achieving enhanced water solubility [8,12,13]. However, only a few studies have addressed the non-covalent functionalization of multi-walled carbon nanotubes (MWCNTs) with dendritic polymers for increased water solubility [14].

Dendritic polymers are highly branched macromolecules of nanosized dimensions, consisting of repeating units and surface end groups [15]. Their properties depend on both the structural characteristics of the branches in their interior, and the large number of surface end groups. These polymers can incorporate a variety of organic compounds as well as inorganic ions in their interior, while the surface end groups are primarily susceptible to functionalization or even multi-functionalization, to yield a variety of novel materials with diversified, tailor-made properties such as drug delivery systems, antibacterial agents, etc. [16,17,18]. Additionally, these terminal groups exhibit the so-called polyvalency effect [19], which enhances their binding with various substrates, due to their close proximity to the dendritic polymers’ scaffold.

Recently, several studies have focused on the potential applications of carbon nanomaterials (CNMs), taking advantage of their antibacterial properties [20,21,22]. Specifically, functionalized single-walled carbon nanotubes (SWCNTs) and multi-walled carbon nanotubes (MWCNTs) were found to exhibit significant antibacterial activity towards both gram-positive and gram-negative bacteria [23,24]. CNTs’ properties, such as carbon nanotube diameter [25], length [26], aggregation [27], concentration [28], surface functionalization [29,30,31], etc. have been reported to influence their antibacterial activity. To that respect, aqueous dispersibility can critically influence antibacterial efficiency, as highly dispersed CNTs enhance their interaction with cells, leading to increased antibacterial properties. Indeed, it was found [32] that individually dispersed CNTs were more toxic to bacteria than CNTs aggregates, due to increased contact with bacterial cells.

Nowadays, there is a growing concern about the possible effects of nanomaterials, as end of life products, on organisms and ecosystems [33,34]. Apart from studies investigating the mechanisms of interaction between CNTs and various biomacromolecules (DNA, RNA, etc.), to identify possible causes of undesired effects, [35] research efforts have also focused on the possible impact of MWCNTs on photosynthetic pathways. Remarkably, in photosynthetic organisms, unlike bacteria, a favorable effect of MWCNTs was observed, e.g., in the development of cereals, and the production of vegetative biomass [36]. Studies on algae revealed that MWCNTs did not influence photosynthesis, and any negative effects were due to turbidity and the resulting reduction of the available light [37]. However, oxidized MWCNTs were found to be toxic to the marine green alga *Dunaliella tertiolecta*, as at concentrations between 1–10 mg/L they reduced algal growth, and affected the Photosystem (PS) II photochemical process and the cellular glutathione redox status [38]. Moreover, although cyanobacterial blooms have become a serious environmental problem, only recently, a novel nanomaterial based on MWCNTs, called Taunit, loaded with antibiotic chloramphenicol or herbicide diuron, was investigated as an effective anti-cyanobacterial agent [39]. It was found that the Taunit-diuron complex exhibited high biocide action against cyanobacterium *Synechocystis* sp. PCC 6803, higher than that of a Taunit-chloramphenicol complex.

In this study, aiming to develop water soluble MWCNTs with enhanced antibacterial/anti-cyanobacteria properties, oxidized multi-walled carbon nanotubes (oxCNTs) were non-covalently functionalized using a series of partially quaternized hyperbranched poly(ethyleneimine) derivatives, yielding novel water-soluble hybrid materials. Specifically, three positively charged derivatives of hyperbranched poly(ethyleneimine) (PEI) with a different degree of quaternization at the primary amino groups of PEI were prepared and interacted with the negatively charged oxidized CNTs through electrostatic interactions and van der Waals attraction forces. The obtained hybrid materials were physicochemically characterized by various techniques (FTIR, Raman, SEM, TEM, AFM, etc.). Their excellent aqueous stability was demonstrated using *ζ*-potential measurements, dynamic light scattering, and UV-vis spectroscopy. Additionally, their antibacterial and anti-cyanobacterial activity was investigated against two types of gram negative bacteria, an autotrophic (cyanobacterium *Synechococcus* sp. PCC 7942) and a heterotrophic (bacterium *Escherichia coli*), while their cytocompatibility was investigated on eukaryotic cell lines.

## 2. Results and Discussion

### 2.1. Synthesis and Characterization of QPEI-Functionalized oxCNTs

**Scheme 1:**
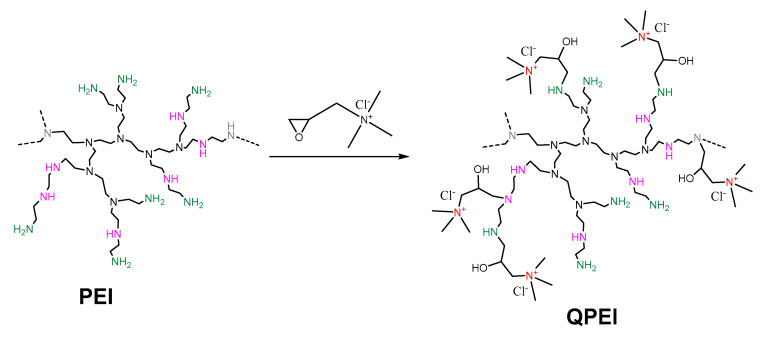
Schematic representation of poly(ethyleneimine) (PEI) and the reaction scheme of quaternization.

Positively charged stable aqueous suspensions of carbon nanotubes were prepared, applying quaternized hyperbranched poly(ethyleneimine) derivatives (QPEIs). A series of partially quaternized hyperbranched poly(ethyleneimine) derivatives with 30%, 50%, and 80% substitution degree of primary amino groups was prepared, following a method analogous to one previously described [40,41]. Initially, PEI was characterized by inverse-gate decoupling ^13^C NMR. According to the literature [42] and comparing the integration of carbons of α-CH_2_ relative to primary, secondary, and tertiary amine groups, the ratio of primary to secondary and to tertiary amines of PEI was found to be 1.00:1.18:1.01. The branching degree was found to be 0.68, and the average number of primary amine groups was determined to be 183. Based on the above, the introduction of α-hydroxyamine moieties, together with the trimethylammonium groups, to PEI was achieved by the reaction of the PEI primary amines with appropriate amounts of glycidyltrimethylammonium chloride, yielding three PEI derivatives, i.e., 30-QPEI, 50-QPEI, and 80-QPEI (Scheme 1). Their structures were confirmed by ^1^H and ^13^C NMR spectroscopy. Specifically, the new quintet appearing at 4.25 ppm was attributed to the proton of α-CH group relative to hydroxyl group, which was formed after an oxiran ring opening. Additionally, the α-CH_2_ protons relative to the quaternary group appeared as a triplet at 3.45 ppm, and the protons of the quaternary methyl groups at 3.25 ppm, while a multiplet in the region 2.50–2.70 ppm was attributed to the PEI scaffold protons. Comparing the integration of peaks at 3.45 ppm and 2.70–2.50 ppm, the degree of quaternization at the primary amino group of PEI was calculated, and was found to be 30%, 50%, and 80% for 30-QPEI, 50-QPEI, and 80-QPEI, respectively.

Furthermore, ^13^C NMR spectroscopy provided insights into the structure of QPEIs. The attachment of the α-hydroxyamine moiety at the PEI scaffold was confirmed by the peaks at 51.0 and 48.0 ppm, attributed to the α carbon of PEI, and relative to the newly formed amino group (α-CH_2_NH-Q), close to secondary (known as C_1,2_) and tertiary (known as C_1,3_) amines, respectively. Additionally, the α-methylene and methyl groups, attached at the quaternary center, were observed at 71.5 and 57.0 ppm, respectively, while a peak at 67.5 ppm was attributed to the α carbon relative to the newly formed hydroxyl group (CH–OH).

**Scheme 2:**
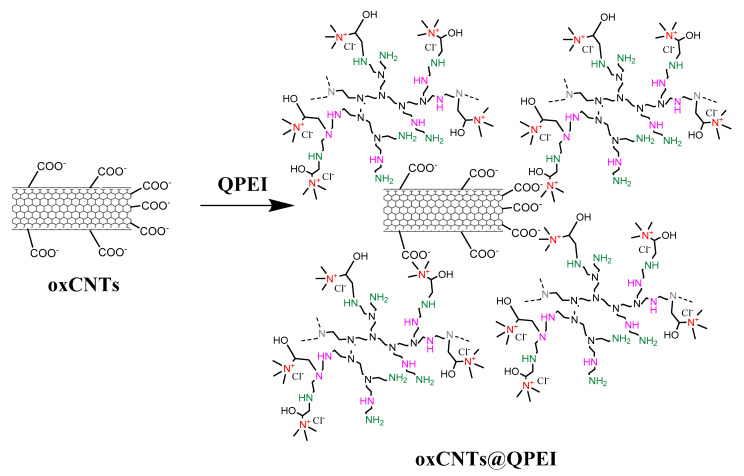
Schematic representation of oxidized multi-walled carbon nanotubes (oxCNTs) decorated with quaternized hyperbranched poly(ethyleneimine) derivative (QPEI).

Subsequently, these dendritic derivatives were interacted with oxidized CNTs in aqueous media (Scheme 2). The final hybrid nanomaterials, oxCNTs@30-QPEI, oxCNTs@50-QPEI, and oxCNTs@80-QPEI, were obtained after ultracentrifugation to remove the excess QPEIs, and physicochemically characterized using a variety of techniques, such as FTIR, RAMAN, TGA, SEM, TEM, AFM, etc.

**Figure 1:**
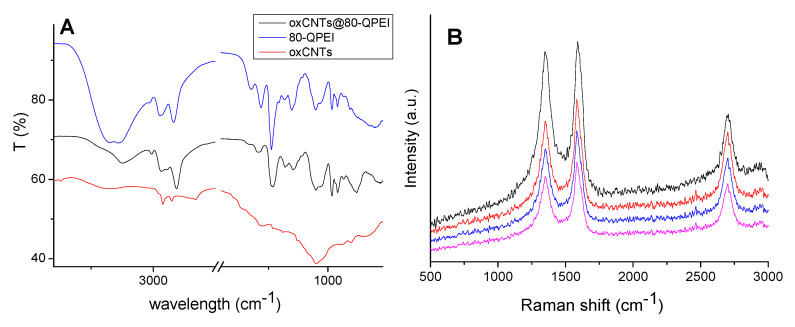
(**A**) FTIR spectra of oxCNTs, oxCNTs@80-QPEI, and 80-QPEI. (**B**) Raman spectra of oxCNTs (black), oxCNTs@30-QPEI (red), oxCNTs@50-QPEI (blue), and oxCNTs@80-QPEI (magenta).

To investigate the successful attachment of QPEIs on the oxCNTs, initially, FTIR spectroscopy was employed. In Figure 1A and Figure S1, the FTIR spectra of QPEIs, oxCNTs, and QPEI-functionalized oxCNTs are shown. The spectrum of oxCNTs shows a C=C stretching band at 1650 cm^−1^, attributed to the CNTs graphite structure. Additionally, the presence of oxygen containing groups (carboxylates, carbonyl, hydroxyl, and epoxy groups) on the oxCNTs was confirmed by the appearance of a C=O stretching band at 1740 cm^−1^, a broad OH stretching band centered at 3370 cm^−1^, and a strong C–OH stretching band at 1100 cm^−1^, as well as two peaks at 1565 and 1380 cm^−1^, which are associated with the carboxylate anion stretch mode (asymmetrical and symmetrical vibrations of COO^-^, respectively). Furthermore, the peaks at 2750 and 1255 cm^−1^, attributed to the stretching vibrations of OC–H and C–O–C, respectively, revealed the presence of aldehydes and epoxy groups on oxCNTs [43]. On the other hand, as expected, the FTIR spectra of all QPEIs exhibited the sample characteristic bands. Those included the characteristic vibrations of PEI, i.e., at 3360 and 3270 cm^−1^, attributed to the stretching vibration of primary and secondary amino groups, at 2940 and 2835 cm^−1^, assigned to the asymmetrical and symmetrical vibrations of CH_2_, at 1600 and 1560 cm^−1^, attributed to the NH deformation mode of the primary and secondary amino groups, respectively, at 1455 and 760 cm^−1^, corresponding to the bending and rocking mode of CH_2_, respectively, and at 1115 and 1050 cm^−1^, assigned to the asymmetrical and symmetrical vibrations of C–N [44]. Additionally, the most important stretching and deformation vibrations of CH_3_ in the quaternary ammonium group (CH_3_)_3_N^+^ at 3030 cm^−1^ and 1485 cm^−1^, the asymmetrical stretching vibration of the whole (CH_3_)_3_N^+^ group at 970 cm^−1^, and the stretching vibrations of C–OH groups at 1100 cm^−1^ were observed [45]. The FTIR spectra of the QPEI-functionalized oxCNTs (Figure 1A and Figure S1) revealed the existence of both oxCNTs and QPEIs, confirming their successful interaction.

The successful functionalization of oxCNTs was also confirmed by Raman measurements. The spectra of oxCNTs and QPEI-functionalized oxCNTs presented in Figure 1B, display the two main typical graphite bands at 1585 cm^–1^ (G band) and 1345 cm^–1^ (D band), attributed to the in-plane vibration of the sp^2^-bonded carbon atoms in graphite layers, and to the defects presented in carbon nanotubes due to the conversion of carbon atoms from an sp^2^ to an sp^3^ hybridization state, respectively. Additionally, a band at ~2700 cm^–1^ (G’ band) is shown in Figure 1B, attributed to the D band overtone. As observed in Figure 1B, the Raman shifts of QPEI-functionalized oxCNTs compared to those of oxCNTs did not change, revealing that the graphitic structure of oxCNTs does not significantly alter after functionalization. Only the value of the intensity ratio of D- to G- bands (I_D_/I_G_), a measure of the defects present in a graphene structure during functionalization, slightly increased from 1.03 to 1.16, revealing successful polymer wrapping all over the graphite layer of CNTs [46]. In analogous studies involving covalent functionalization of multi-walled carbon nanotubes via third-generation dendritic poly(amidoamine) or amphiphilic poly(propyleneimine) dendrimers, a larger increase of the intensity ratio (I_D_/I_G_), was reported indicating that functionalization caused a larger defect of the graphitic network [47,48]. In contrast, in the present study oxCNTs were non-covalently functionalized with QPEI derivatives, retaining their surface almost intact.

**Figure 2:**
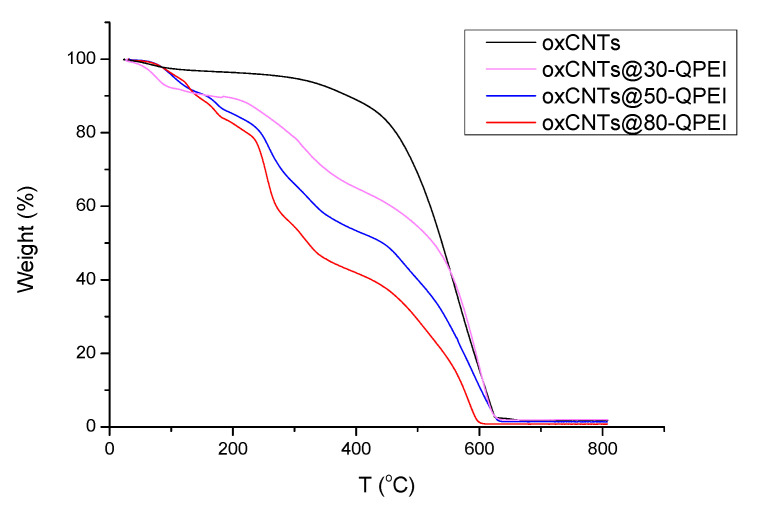
Thermogravimetric curves of oxCNTs and QPEI-functionalized oxCNTs.

The results of thermogravimetric analysis (TGA) provided further information on the QPEI content on the surfaces of oxCNTs (Figure 2). In the TGA curve of oxCNTs, two distinct decomposition regions were observed. Specifically, a weight loss corresponding to ~4% of the initial weight was recorded in the temperature range of 180–400 °C, due to the removal of oxygen-containing functional groups present on the graphitic framework. A second significant loss was observed at higher temperatures (>500 °C), and was attributed to the thermal degradation of the graphitic framework. In contrast, the QPEI-functionalized oxCNTs exhibited a significant weight loss (10–20%) up to 250 °C, due to both the removal of oxCNTs’ oxygen-containing groups, and the partial PEI degradation. The weight loss for QPEI-functionalized oxCNTs in the temperature range 250–400 °C was significantly higher in comparison to oxCNTs (up to 60%), since together with the decomposition of graphitic lattice, QPEI molecules were removed from the graphitic framework. Therefore, TGA measurements provided further qualitative experimental evidence that functionalization had occurred, however, due to the oxCNT contribution to the thermal phenomena, the polymer content could not be quantified. Again, above ~500 °C, the sharp weight loss indicated the total thermal destruction of the graphitic network.

Elemental analysis measurements were additionally performed to confirm and quantify the polymer content in the QPEI-functionalized oxCNTs. Given that the nitrogen signal in the final hybrid originated mainly from QPEI, the difference compared to the starting oxCNTs represented the amount of polymer attached to the CNTs. Therefore, the QPEI content in hybrids was calculated from the following formula:

QPEI (% *w*/*w*) = (*N*_s_ − *N*_CNTs_)/(*N*_QPEI_ − *N*_CNTs_) × 100

where *N*_s_, *N*_QPEI,_ and *N*_CNTs_, were the nitrogen elemental mass fraction in QPEI-functionalized oxCNTs, QPEI, and oxCNTs, respectively [49]. The results are summarized in Table S1. According to the elemental analysis results, the actual value of QPEI weight fraction in oxCNTs@30-QPEI, oxCNTs@50-QPEI, and oxCNTs@80-QPEI was found to be 16.05%, 19.92%, and 23.23%, respectively.

**Figure 3:**
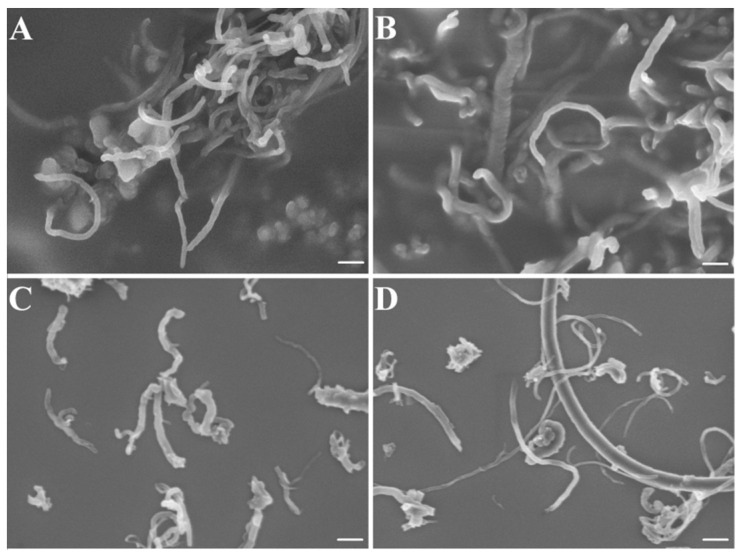
SEM images of oxCNTs@30-QPEI (**A**), oxCNTs@50-QPEI (**B**), and oxCNTs@80-QPEI (part **C,D**). The scale bar is 100 nm.

The morphology of the QPEI-functionalized oxCNTs was studied by scanning electron (SEM), transmission electron (TEM), and atomic force (AFM) microscopies. Representative SEM micrographs are shown in Figure 3. It is clear that after functionalization with QPEIs, the morphology of the oxCNTs did not change significantly. Additionally, oxCNTs@QPEIs are shown to be well-dispersed and no aggregation of nanotubes was observed, as in the case of oxCNTs (Figure S2). Especially, functionalization of oxCNTs with 80-QPEI rendered them fully isolated, as shown in Figure 3 (images C and D).

**Figure 4:**
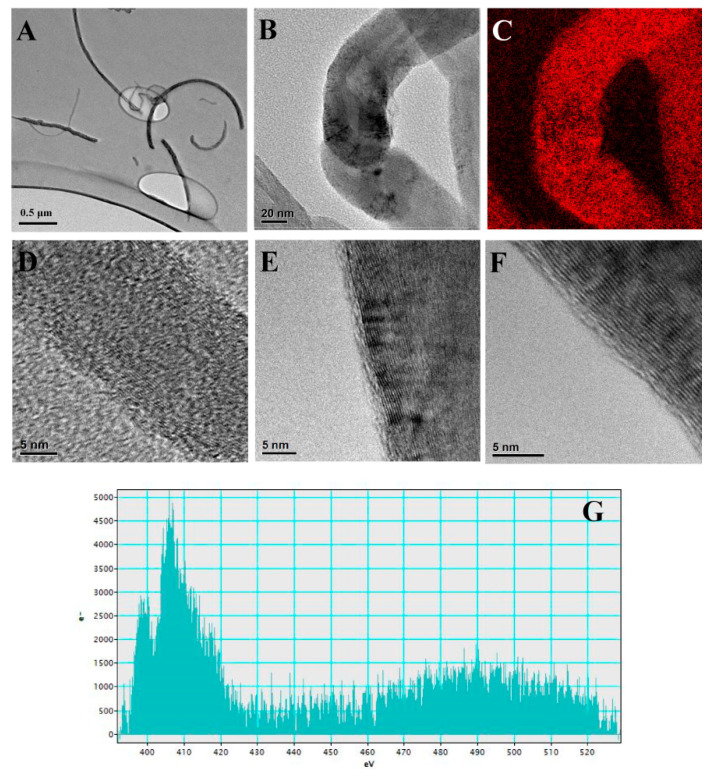
TEM bright field image of oxCNTs@50-QPEI (**A**). Bright field image (**B**) and the corresponding EFTEM compositional nitrogen N map (red, **C**) of oxCNTs@50-QPEI, HRTEM images: images of oxCNTs@80-QPEI (**D**–**F**) and a typical background subtracted EELS spectrum of nitrogen K- edges, recorded for oxCNTs@80-QPEI (**G**).

The morphology of QPEI-functionalized oxCNTs, as well as the presence of QPEIs on their surface, was studied by combining TEM bright-field imaging, EFTEM elemental mapping, and EELS spectroscopy. In Figure 4A functionalized carbon nanotubes are observed isolated, without any aggregation, as in the SEM images. These observations suggest that the QPEIs covered the surface of the nanotubes, improving their aqueous dispersibility and debundling. In the HRTEM images the structured graphite walls of oxCNTs can be observed, covered with an amorphous layer of QPEI polymer (Figure 4D,E). For this reason, electron energy loss spectroscopy (EELS) was employed to investigate the spatial distribution of nitrogen, observed in the bright field images of oxCNTs. An energy-filtered TEM (EFTEM) image (utilizing the three-window method), using the nitrogen K-edge at 401 eV electron energy loss, can be seen in Figure 4C, while Figure 4B is the bright field image of the same area. It is obvious that since the intensity of the maps corresponds to the concentration of N (red) that exclusively originated from QPEI, it can be concluded that oxCNTs were uniformly covered by QPEI. Additionally, in a typical background subtracted EELS spectrum the nitrogen K-edges recorded for oxCNTs@80-QPEI (Figure 4) are evidence for the presence of nitrogen atoms and the successful attachment of QPEI on the surface of oxCNTs.

**Figure 5:**
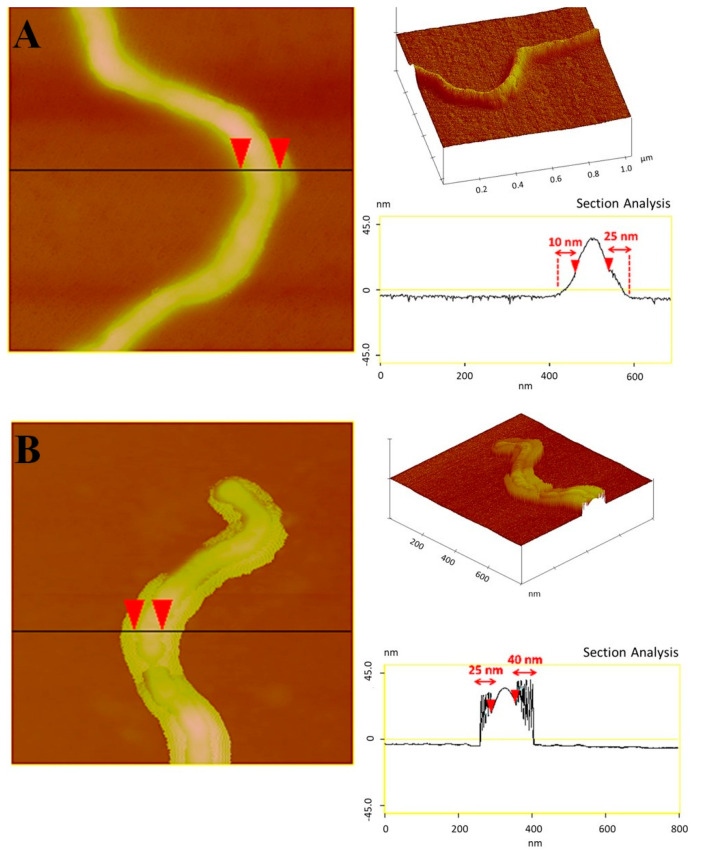
AFM images (height, profile section analysis, and 3D) of oxCNTs@50-QPEI (**A**) and oxCNTs@80-QPEI (**B**), showing the coverage of QPEI derivatives in the sidewalls of oxCNTs.

AFM images of oxCNTs@50-QPEI and oxCNTs@80-QPEI, deposited on Si-wafer (Figure 5) show the morphological features of oxCNTs at the nanoscale after the interaction with the polymers. The AFM images (height and 3D) of nanocomposites reveal the successful attachment (wrapping) of polymer on the oxCNTs sidewalls. As derived from topographical section analysis, an overlay of 10–25 nm is observed from each side of nanotube in the case of oxCNTs@50-QPEI, while the size of the polymeric coating is much higher, and easily distinguishable in the case of oxCNTs@80-QPEI, corresponding to an average of 25–40 nm (Figure 5). Moreover, the average diameter of oxCNTs@80-QPEI, as derived from height analysis, is about 40–50 nm, a value much higher than that of oxCNTs in the absence of 80-QPEI, which ranges between 15 and 25 nm (Figure S2).

### 2.2. Colloidal Stability of the CNTs Dispersions

**Figure 6:**
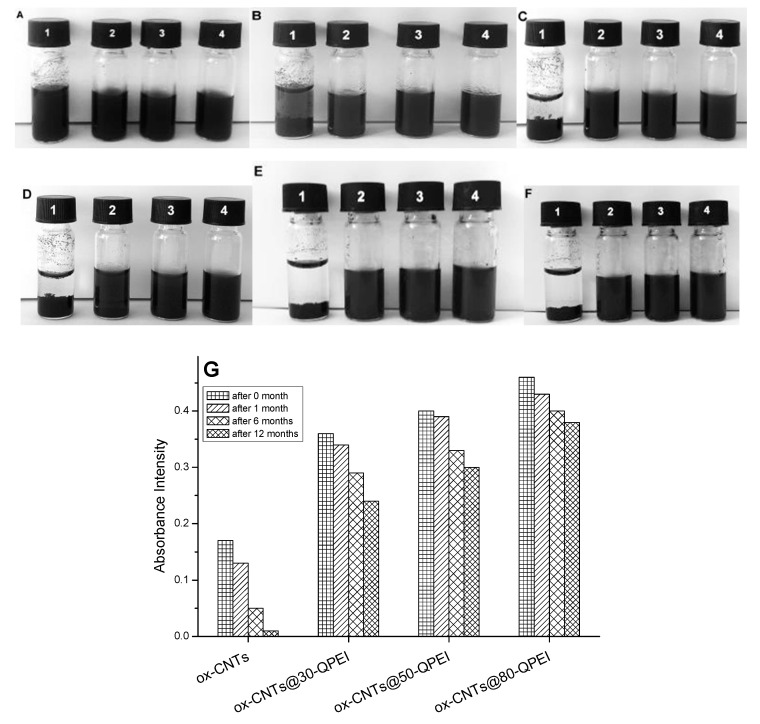
Dispersion state of (1) oxCNTs, (2) oxCNTs@30-QPEI, (3) oxCNTs@50-QPEI, and (4) oxCNTs@80-QPEI in water (5 mg/mL), (**A**) immediately after sonication, and after quiescent settling for (**B**) 2 weeks, (**C**) 1 month, (**D**) 3 months, (**E**) 6 months, and (**F**) 12 months (upper part). (**G**) Sedimentation behavior of oxCNTs and QPEI-functionalized oxCNTs at different aging times (lower part).

CNTs have an extremely strong tendency to aggregate in water due to their high surface energy, making them difficult to disperse in aqueous media resulting in the formation of large bundles [50]. Although the dispersibility of CNTs in aqueous media has been shown to increase following (i) various oxidation processes [51], and (ii) using high concentrations of various surfactants [6,7] or polymers [8,9], the resulting dispersions are only stable for short time. In this study, the negatively charged oxidized CNTs, modified with positively charged QPEIs through electrostatic interactions and van der Waals attraction forces, resulted in functionalized oxCNTs with high positive charge contents, and able to form stable aqueous dispersions. All QPEIs derivatives enhance the aqueous dispersibility of oxCNTs, as revealed by visual observation over time (Figure 6, upper part). It is obvious that stable dispersions of oxCNTs were obtained after functionalization with QPEIs for at least twelve months (Figure 6, upper part), while oxCNTs had precipitated within one month after the sonication process. This was achieved thanks to the presence of quaternary ammonium groups on the surface of the oxCNTs that provide a high compatibility with aqueous media due to their strong hydrophilicity, while preventing the CNTs’ aggregation due to electrostatic repulsion. In an analogous study, involving SWCNTs, Grunlan, J.C. et al. observed that after non-covalent functionalization with PEI, SWCNTs exhibited poor aqueous stability, attributed to PEIs hyperbranched structure that sterically reduced electrostatic interactions [52]. The same behavior was observed in this study for PEI functionalized oxCNTs. In contrast, quaternized PEI derivatives behave differently, probably due to their higher positively charged moieties content.

It is known that bundled CNTs, unlike individual ones, are not active in the UV–vis region [53] allowing the investigation of their dispersibility using UV–vis absorption spectroscopy. Figure S3 shows the UV–vis spectra of the oxCNTs@30-QPEI, oxCNTs@50-QPEI, and oxCNTs@80-QPEI aqueous dispersions. The dispersions have a characteristic absorption peak at 263 nm, affected by the p-plasmon absorption of carbon nanomaterials. The higher absorption, caused by the p-plasmon from the oxCNTs@80-QPEI, demonstrates more efficient debundling of oxCNTs by 80-QPEI, compared to other carbon nanomaterials [54]. Furthermore, the evaluation of colloidal stability of the CNTs was attained by UV–vis spectroscopy, again after investigation of the characteristic absorption of CNTs at 263 nm. Figure 6G presents the optical density (O.D.) of the oxCNTs and QPEI-functionalized oxCNTs dispersed in water within the storage periods. It was obvious that the dispersions of all QPEI-functionalized oxCNTs were stable for at least twelve months, since their optical densities were reduced only by 10–20% compared to the initial O.D. On the other hand, the O.D. of the oxCNTs dispersion was reduced by 90% after 12 months storage. The O.D. reduction of oxCNTs dispersion was attributed to the gradual formation of CNTs agglomerates, some of which subsequently aggregated and finally precipitated. These findings are in line with the visual inspection of the CNT dispersions presented in Figure 6A–F. Moreover, the dispersion of oxCNTs@80-QPEI is the most stable since only a 10% reduction of O.D. was observed after 12 months storage. To the best of our knowledge the stability achieved is one of the highest reported in the literature, and its importance lies in the fact that the aqueous stability of such hybrid nanomaterials is of paramount importance in several industrial applications.

### 2.3. Characterization of the QPEI-Functionalized oxCNTs Dispersions

**Figure 7:**
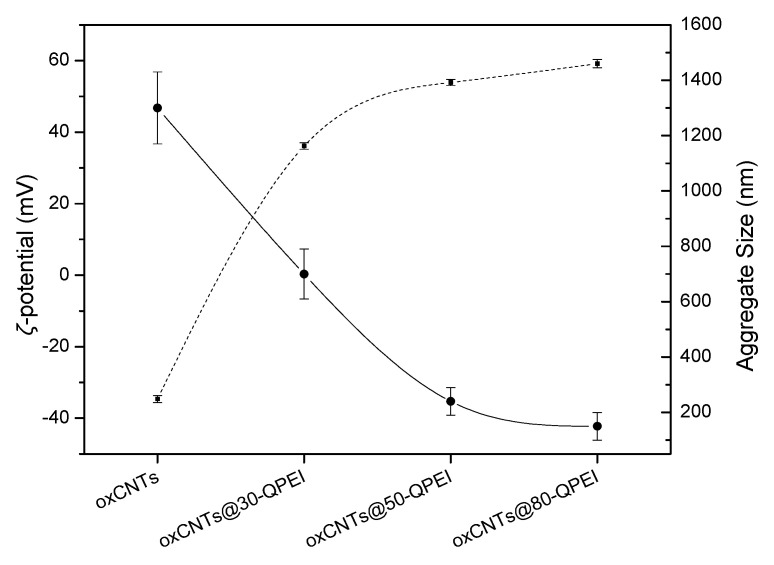
Mean hydrodynamic diameter (solid line) and *ζ*-potential values (dot line) of oxCNTs and QPEI-functionalized oxCNTs dispersions (0.05 mg/mL, pH = 7.0).

Dynamic light scattering (DLS) and *ζ*-potential measurements can provide information on nanomaterials regarding their size distribution, and also their surface charge. Although, DLS measurement is appropriate for determination of the spherical particle diameter, it can also be used to determine the hydrodynamic diameter of nanotubes, based on the assumption that an equivalent hydrodynamic diameter (D*_h_*) of a sphere has the same diffusion properties as the CNT. [55] Even though one cannot determine absolute values, a relative size comparison can be obtained for similarly shaped materials [56]. Thus, comparing the aggregate size of oxCNTs to those of QPEI-functionalized oxCNTs, it is obvious that debundling of the oxCNTs took place after their interaction with the QPEIs, since the value of hydrodynamic diameter of the oxCNTs decreased from 1300 nm to 150 nm when 80-QPEI was used (Figure 7).

Zeta potential values of the oxCNTs dispersions at pH = 7.0 are given in Figure 7. As expected, the aqueous dispersion of oxCNTs has a *ζ*-potential value around −34 mV, due to their negative surface charges. After modification with QPEI, the *ζ*-potential values of the QPEI-functionalized oxCNTs dispersions increased to positive, reaching the value of +60 mV for oxCNTs@80-QPEI, offering further evidence that the positively charged QPEIs were successfully attached onto the oxCNTs surface. It should be noted that all *ζ*-potential values were higher than +30 mV, indicating stable aqueous colloidal suspensions, in which the CNTs’ aggregation was prevented due to electrostatic repulsion, in line with the results of UV–vis and DLS measurements (see above) [57].

### 2.4. Evaluation of Antibacterial and Anti-Cyanobacterial Activity

The cytotoxicity of QPEI-functionalized oxCNTs was assessed against two types of Gram-negative bacteria, i.e., the heterotrophic bacterial strain *Escherichia coli* XL1-blue and the autotrophic cyanobacterium *Synechococcus* sp. PCC 7942.

#### 2.4.1. Cytotoxicity Effects of oxCNTs@QPEIs on *Escherichia coli* XL1-Blue Bacteria

**Figure 8:**
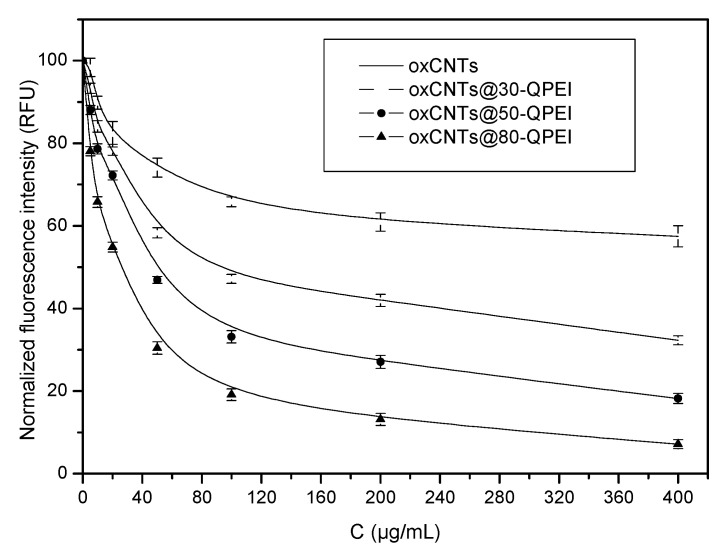
Cytotoxicity of oxCNTs and QPEI-functionalized oxCNTs against gram-negative *Escherichia coli* XL1-blue bacteria. Fluorescence intensity of RFP at 590 nm (excitation: 545 nm) after bacteria incubation for 6 h with oxCNTs@QPEIs at concentrations ranging from 5 to 400 µg/mL, normalized with the initial fluorescence intensity, corresponding to initial *E.coli* population (at OD_600_ = 0.4). Error bars represent mean ± SD for at least three independent experiments.

*Escherichia coli* growth was investigated by monitoring the fluorescence intensity of bacterial cells suspensions at 37 °C that express red fluorescent protein (RFP). The excellent dispersibility of oxCNTs@QPEIs renders the commonly used turbidity measurement inapplicable, as functionalized CNTs, especially at high concentrations, contribute to the final measurement. Thus, by employing the inherent fluorescence of RFP, the antibacterial activity can be precisely assessed, even in the presence of nanoparticle dispersions, as in the case of oxCNTs@QPEIs. Comparing the fluorescence intensity of each bacterial suspension containing oxCNTs@QPEIs at a certain time, with the initial fluorescence intensity corresponding to the initial *Escherichia coli* population (at OD_600_ = 0.4), bacterial growth could be directly determined. Figure 8 depicts the *Escherichia coli* growth after 6 h incubation in the presence of oxCNTs and oxCNTs@QPEIs at different concentrations, ranging from 5 to 400 µg/mL, as a function of the fluorescence intensity of RFP at 590 nm (excitation: 545 nm), and normalized with the initial fluorescence intensity of control (100% fluorescence intensity). Contrary to the increase of the fluorescence intensity upon untreated bacteria growth (Figure S4), a decrease in the intensity was observed, for all samples, revealing bacterial growth inhibition. However, as shown in Figure 8, the oxCNTs exhibited low antibacterial activity, which is in accordance with the literature [58]. On the other hand, all QPEI-functionalized oxCNTs inhibited bacterial growth in a dose-dependent manner, displaying significantly higher antibacterial activity than the oxCNTs, and which increased upon substitution of PEI from 30% to 80%. In a further attempt to quantify the antibacterial activity of oxCNTs@QPEIs, the 50% inhibitory concentrations (IC-50) were calculated (Table 1). It was found that the lowest IC-50 was observed in the case of oxCNTs@80-QPEI (28.4 μg/mL), which showed that oxCNTs@80-QPEI exhibited the highest antibacterial activity amongst the other hybrid materials, due to both the increased aqueous dispersibility and the higher positive quaternary ammonium group content.

**Figure 9:**
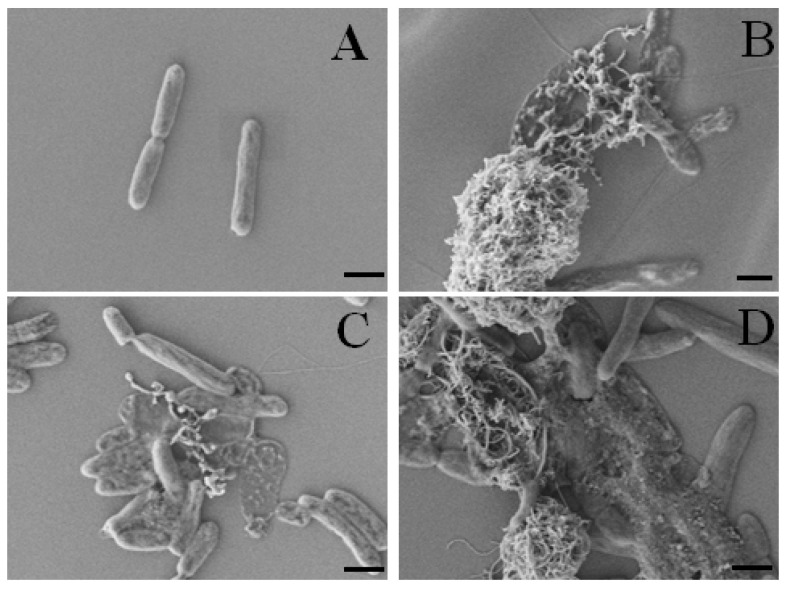
SEM images of *Escherichia coli* bacteria: untreated cells (**A**) and cells after 6-h incubation time at 37 °C with oxCNTs@30-QPEI (**B**), oxCNTs@50-QPEI (**C**), and oxCNTs@80-QPEI (**D**) at 50% inhibitory concentrations. The scale bar is 1 μm.

The morphology of *Escherichia coli* after 6-h incubation at 37 °C with oxCNTs@QPEIs was investigated by scanning electron microscopy (SEM). In Figure 9, SEM images of control (untreated cells) and cells treated with oxCNTs@QPEIs at 50% inhibitory concentrations are presented, showing significant changes in cell morphology. Specifically, the treated cells lost their cellular integrity, and are shown more clustered, while their cell walls seem rougher and damaged in all cases (Figure 9B–D) compared to the untreated cells (Figure 9A), which appear to be intact, with a smooth surface. Moreover, Figure 9D shows the most severe effect of oxCNTs@80-QPEI on the bacterial cell wall and membrane, in which the cell walls seem to be ruptured and bacterial cell lysis is clearly observed probably due to membrane damage. 

It is known that highly dispersed carbon nanotubes, mainly single wall carbon nanotubes, are able to interact strongly with bacteria through van der Waals forces, forming bacteria-CNTs aggregations [20,25]. This fact results in bacterial death due to either inhibition of transmembrane electron transfer, or to penetration leading to rupture or deformation of cell walls and membranes, which alter the bacterial metabolic processes [59]. Moreover, SWCNTs and MWCNTs containing various types of surface groups were investigated [29] regarding their antibacterial activity towards gram-negative and gram-positive bacteria. It was found that SWCNTs functionalized with hydroxyl and carboxyl surface groups exhibited improved antimicrobial activity against both gram-positive and gram-negative bacteria. However, MWCNTs containing the same functional surface groups did not exhibit any significant antibacterial effect [29]. On the contrary, covalently functionalized MWCNTs with positive moieties such as amines, arginines, and lysines, [60,61] or MWCNTs combined with surfactant molecules, such as dioctyl sodium sulfosuccinate [32], hexadecyltrimethylammonium bromide, triton X-100, and sodium dodecyl sulfate [7], exhibited enhanced antibacterial properties, due to enhanced interactions with bacterial membranes and the improved aqueous dispersibility and stability. In this study, similar antibacterial behavior of QPEI-functionalized oxCNTs was observed due to the high positive quaternary ammonium group content. These positive groups, as in the case of surfactant molecules or other positive functional groups, not only improved the debundling of MWCNTs, which favors the strong interaction between the bacteria and MWCNTs, but also enhanced the penetration of MWCNTs though cell membranes, resulting in cell lysis and death.

Moreover, it is known that the quaternary ammonium moieties efficiently interact with the negatively charged groups of bacterial walls or cytoplasmic membranes, mainly through electrostatic as well as with secondary hydrophobic interactions, leading to dysfunction in cellular processes and probably to cell death [62]. Highly functionalized polymers with quaternary ammonium groups have been found to be more effective antibacterial agents than their low molecular weight analogues, as their higher charge densities lead to stronger interactions with the negatively charged bacteria walls [63]. For example, poly(propylene imine) dendrimers bearing 16 quaternary ammonium groups per molecule were found to exhibit two orders of magnitude greater antimicrobial activity than their mono-functional counterparts [64]. In agreement with this, the 80-QPEI derivative containing the highest content of quaternary ammonium groups provided the highest polycationic character to oxCNTs, in regards to the other QPEI derivatives. This effect probably induces the strongest interaction with bacteria, and the highest permeability of the cell membrane, and thus oxCNTs@80-QPEI exhibited the best antibacterial activity against the *Escherichia coli* bacteria compared to the other hybrid materials.

#### 2.4.2. Cytotoxicity Effects of oxCNTs@QPEIs on *Synechococcus* sp. PCC 7942 Cyanobacteria

The antibacterial activity of oxCNTs@QPEIs was further assessed against the cyanobacterium *Synechococcus* sp. PCC 7942, a very widespread bacterial strain in the aquatic environment. In general, cyanobacteria (gram negative bacteria) are prokaryotic organisms that perform oxygenic photosynthesis similar to higher plants. They are the oldest and one of the largest and most important groups of bacteria on earth. Cyanobacteria (except prochlorophytes) contain only Chl *α*, the molecule which makes photosynthesis possible, by passing its energized electrons on to molecules during sugar synthesis [65]. However, several cyanobacterial strains are known to produce a wide range of toxic secondary metabolites (hepatotoxins, neurotoxins, cytotoxins, dermatotoxins, and irritant toxins), which could be harmful to animals and potentially dangerous to humans [66].

**Figure 10:**
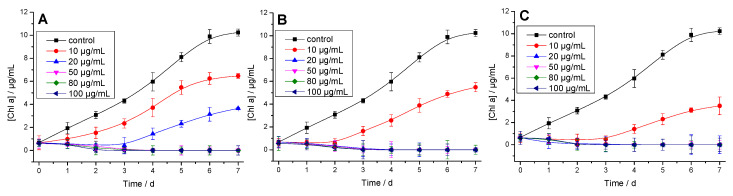
Effect of oxCNTs@QPEIs on cell proliferation of cyanobacteria *Synechococcus* sp. PCC 7942. Growth curves of cyanobacteria in the presence of different concentrations of: oxCNTs@30-QPEI (**A**), oxCNTs@50-QPEI (**B**), and oxCNTs@80-QPEI (**C**). Error bars represent mean ± SD for at least three independent experiments.

In this study, cyanobacteria *Synechococcus* sp. PCC 7942 cell proliferation was monitored by measurement of the Chl *α* concentration every 24 h, for seven days. Figure 10 shows the cell proliferation of the unicellular cyanobacterium *Synechococcus* sp. PCC 7942 cells in the presence of increasing concentrations of oxCNTs@30-QPEI, oxCNTs@50-QPEI, and oxCNTs@80-QPEI. For comparison reasons, analogous experiments were performed using oxCNTs, and their effect on the cyanobacteria cell proliferation is shown in Figure S5. It is clear that oxCNTs did not inhibit the cyanobacterial cell proliferation in contrast to all oxCNTs@QPEIs. This can be attributed again to the strong positive character of oxCNTs@QPEIs that intensified the interaction with the cyanobacteria membrane, and resulted in higher cell penetration compare to oxCNTs. In order to quantify these results, the effective concentration for 50% inhibition (IC-50), which shows the ability of cells to proliferate under the toxic effect of the oxCNTs@QPEIs, was calculated from the cyanobacterial cell proliferation curves in the presence of each hybrid material (Figure S6 and Table 2). Based on the interpretation of the experimental data using a non-linear regression of the four-parameter logistic function (Figure S6), it is obvious that cell proliferation is concentration dependent, while oxCNTs@80-QPEI exhibited the most promising anti-cyanobacterial activity, compared to the other two hybrid materials. This implies that upon increasing the degree of quaternization, the proliferation rate of cyanobacteria cells decreases. Therefore the anti-cyanobacterial properties, as in case of *Escherichia coli*, depend, not only on the concentration, but also on the degree of quaternization.

Triggered by these results, it was interesting to evaluate the effect of oxCNTs@QPEIs on the photosynthetic apparatus of cyanobacteria. Therefore, the activity of Photosystem (PS) I and II was assessed in the presence of oxCNTs@80-QPEI, the material exhibiting the best antibacterial performance, to investigate the consequences of oxCNTs@QPEIs on the integrity of the photosynthetic apparatus in terms of photoinduced electron transport.

**Figure 11:**
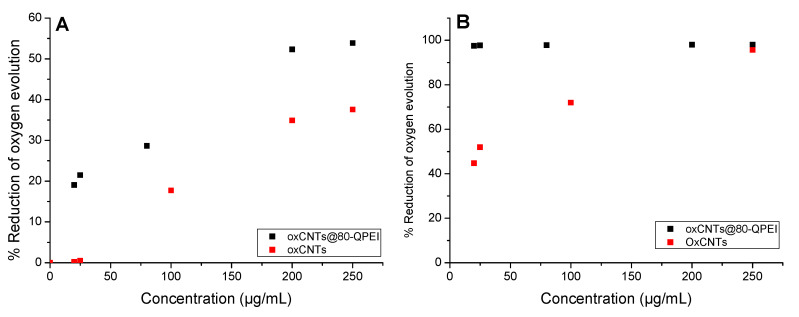
Effect of oxCNTs and oxCNTs@80-QPEI on the photosynthetic electron transport activities of PSII (**A**) and PSI (**B**) in *Synechococcus* sp. PCC 7942 permeaplasts.

Specifically, selective detection of the PSII [67] and PSI electron transporting activities [68] was performed on *Synechococcus* sp. PCC 7942 bacteria treated with lysozyme (permeaplasts) at room temperature [69]. Using *Synechococcus* permeaplasts, oxymetrically photoinduced electron transport activities, across both PSII (electron donor: water; post-PSII electron acceptor: p-benzoquinone) and PSI (post- PSII inhibitor: DCMU; post-PSII electron donor: diaminodurene and ascorbate; post-PSI electron acceptor: methyl viologen) were measured. It was found that upon increase in concentration of oxCNTs@80-QPEI, the rate of oxygen evolution decreases (Table S2), indicating that PSII and PSI electron transport activities depend on the QPEI-functionalized oxCNT concentration. The inhibition of PSI and PSII by oxCNTs and oxCNT@80-QPEI is shown in Figure 11. Similarly to results previously reported in the literature, [70], at high concentrations (250 μg/mL) the oxCNTs used in this study inhibited the PSII and PSI by 37.6% and 95.7%, respectively, while at lower concentrations (20 μg/mL) the PSII is practically unaffected and the PSI activity is reduced by almost 44.8%. However, the impact of oxCNT@80-QPEI was much higher. The novel hybrid with an 80% quaternization degree inhibited the PSII by 16.7% at concentration 20 μg/mL, and by around 53.9% at 250 μg/mL. In the case of the PSI, the effect was even more significant, exhibiting almost complete inhibition (more than 97%), even at low concentrations (20 μg/mL).

The remarkable decrease in the IC-50 values of oxCNTs@QPEIs on cyanobacterial cell proliferation (Table 2) indicates that photosynthetic electron transport of both PSII and PSI is functionally impaired in cyanobacterial cells. Although, oxCNTs inhibit the photosynthetic redox reactions, and in the case of PSI almost fully prevent its activity, the effect of QPEI is significant. This may be attributed to the polycationic character of QPEI, as such compounds are known to completely inhibit PSI reactions, while leaving PSII relatively unaffected [71]. However, under the test conditions oxCNTs did not inhibit cyanobacterial cell proliferation (Figure S5), implying that the oxCNTs could not penetrate the cyanobacteria membranes. On the contrary, as mentioned above, the oxCNTs@QPEIs, exhibited high toxicity against cyanobacteria as a result of their efficient cell penetration (Figure 10).

Furthermore, to elucidate the potential alternative patterns for electron flow to and from PSI, the P700^+^ transients in the presence of oxCNTs@80-QPEI were investigated. Table S3 shows the amounts of functional PSI complexes, estimated as the photooxidizable form of the PSI (P700^+^) reaction center, measured as ΔA820/A820 [72], where an 80% inhibition by oxCNTs@80-QPEI at high concentrations (250 μg/mL) can be observed. In a previous study, MWCNTs were successfully applied for direct transfer of electrons in isolated spinach thylakoids and cyanobacteria Nostoc sp. [73], pointing out that the results obtained in this study may also be associated with an interruption of the electron transport, due to the presence of oxCNTs. Concomitantly, the lower steady state photooxidation of P700 by far red light, might also be considered as an indication that oxCNTs@QPEIs quench the P700^+^. Based on the above, the observed enhanced anti-cyanobacterial effect of QPEI-functionalized oxCNTs on cyanobacterium *Synechococcus* sp. PCC 7942 is ascribed to the selective inhibition of PSI.

### 2.5. Cell Viability Assay

**Figure 12:**
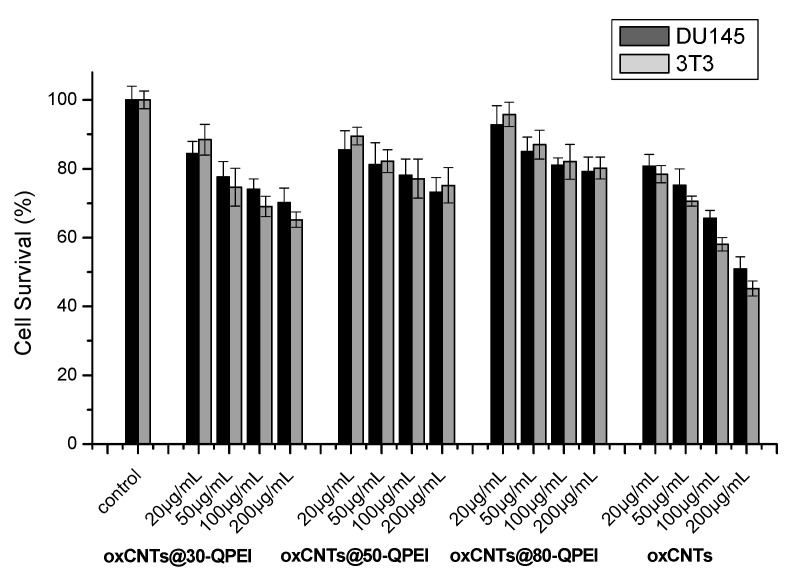
Cytotoxicity of oxCNTs and QPEI-functionalized oxCNTs on DU145 and 3T3 cells following incubation at various concentrations for 24 h, as determined by MTT assays. Data are expressed as mean ± SD of eight independent values obtained from at least three independent experiments.

To investigate the cytotoxicity of QPEI-functionalized oxCNTs, the human prostate carcinoma DU145 cell line and the 3T3 mouse fibroblasts were employed. For this purpose, these cells were incubated for 24 h with oxCNTs and oxCNTs@QPEIs at concentrations below and above their IC-50 values and cell viability was assessed, employing the standard MTT assay. The percent cell viability caused by all derivatives is presented in Figure 12. It is obvious that all oxCNTs@QPEIs were not toxic at their IC-50 values, and significantly less lethal than the parent oxCNTs, while at higher concentrations (100–200 μg/mL) only oxCNTs@30-QPEI exhibited slight toxicity (~70% cell survival). It should be noted that at 200 μg/mL, a concentration much higher than their IC-50 values, oxCNTs@50-QPEI and oxCNTs@80-QPEI did not display any cytotoxicity. These results suggest that all oxCNTs@QPEIs simultaneously exhibited both low cytotoxicity and enhanced antibacterial/anti-cyanobacterial properties.

## 3. Materials and Methods

### 3.1. Chemicals and Reagents

Hyperbranched poly(ethyleneimine) (PEI) with molecular weight 25 KDa (Lupasol^®^ WF, water-free, 99%) and oxidized multi-walled carbon nanotubes were kindly donated by BASF (Ludwigshafen, Germany) and Glonatech S.A. (Athens Greece), respectively. Glycidyltrimethylammonium chloride, dialysis tubes (molecular weight cut-off: 1200) and triethylamine were obtained from Sigma-Aldrich (St. Louis, MA, USA). D-MEM low glucose with phenol red, l-glutamine, phosphate buffer saline (PBS), fetal bovine serum (FBS), penicillin/streptomycin, and trypsin/EDTA were purchased from BIOCHROM (Berlin, Germany). Thiazolyl blue tetrazolium bromide (MTT) and isopropanol were purchased from Merck KGaA (Calbiochem^®^, Darmstadt, Germany).

### 3.2. Synthesis of Quaternized Hyperbranched Poly(ethyleneimine) Derivatives

Quaternized derivatives of hyperbranched poly(ethyleneimine), with different substitution degrees of primary amino groups were prepared by a method previously described [40,41]. In brief, to an aqueous solution (20 mL) of PEI (5 mM), an aqueous mixture (10 mL) containing, 8, 12, or 18 mmol glycidyltrimethylammonium chloride and 16, 24, or 36 mmol triethylamine, respectively, was added. The reaction was completed after two days at room temperature and the final quaternized derivatives with 30% (30-QPEI), 50% (50-QPEI), and 80% (80-QPEI) degree substitution of primary amino groups were received after dialysis against deionized water and lyophilization. The introduction of the quaternary moieties at the external surface of the parent PEI was confirmed by ^1^H and ^13^C NMR spectroscopy. Additionally, the degree of quaternization at the primary amino groups of PEI was calculated by the integration of peaks at 3.45 ppm and 2.70–2.50 ppm in the ^1^H NMR spectra.

^1^H NMR: (500 MHz, D_2_O) *δ* (ppm) = 4.25 (broad s, CH–OH), 3.45 (m, CH_2_N^+^(CH_3_)_3_), 3.25 (s, CH_3_), 2.70–2.50 (m, CH_2_ of PEI scaffold).

^13^C NMR (125.1 MHz, D_2_O): *δ* (ppm) = 71.5 (CH_2_N^+^CH_3_)_3_), 67.5 (CH–OH), 57.0 (CH_3_), 55–51.0 (CH_2_ of PEI scaffold), 51.0 and 48.0 (CH_2_NH-Q primary and secondary, respectively), 42.0 and 40.0 (CH_2_NH_2_ close to secondary (C_1,2_) and tertiary (C_1,3_) amine, respectively).

### 3.3. Preparation of QPEI-Functionalized oxCNTs

The functionalization of oxCNTs was achieved by a method previously described [41]. Specifically, 50 mg of oxCNT powder was dispersed in 50 mL of an aqueous solution, containing an excess quantity (150 mg) of each quaternized derivative, and the resulting dispersions were ultrasonicated for 15 min (Hielscher UP200S high intensity ultrasonic processor equipped with a standard sonotrode (3 mm tip-diameter) at 50% amplitude and 0.5 cycles) and stirred for a further 12 h, at room temperature. The final hybrid materials, oxCNTs@30-QPEI, oxCNTs@50-QPEI, and oxCNTs@80-QPEI, were received after ultracentrifugation at 45,000 rpm, followed by thorough washing with water to remove the unreacted QPEI derivatives and lyophilization.

### 3.4. Characterization of QPEI-Functionalized oxCNTs

FTIR spectra were recorded using a Nicolet 6700 spectrometer (Thermo Scientific, Waltham, MA, USA) equipped with an attenuated total reflectance accessory with a diamond crystal (Smart Orbit, Thermo Electron Corporation, Madison, WI, USA). Raman spectra were obtained using a micro-Raman system RM 1000 Renishaw (laser excitation line at 532 nm, Nd-YAG) in the range of 400–2000 cm^−1^. AFM images were obtained in tapping mode, with a 3D Multimode Nanoscope, using Tap-300G silicon cantilevels with a <10 nm tip radius and a ≈20–75 N/m force constant. Samples were deposited onto silicon wafers (P/Bor, single side polished) by drop casting from ethanol solutions. Scanning electron microscopy (SEM) images were recorded using a Jeol JSM 7401F field emission scanning electron microscope equipped with a gentle beam mode. Transmission electron micrographs were taken using a Philips C20 TEM instrument equipped with a Gatan GIF 200 energy filter for electron energy loss elemental mapping. For the sample preparation, a drop of oxCNTs@QPEIs aqueous solution (0.1 mg/mL) was casted on a PELCO^®^ Formvar grid and was left to evaporate. Thermogravimetric analyses (TGA) were carried out on a Setaram SETSYS Evolution 17 system at a 5 °C/min heating rate under oxygen atmosphere. Elemental analysis (EA) was measured by a Perkin Elmer 240 CHN elemental analyzer.

### 3.5. Preparation and Characterization of QPEI-Functionalized oxCNTs Aqueous Dispersions

The suspensions of polymer-functionalized oxCNTs were prepared by adding 10 mg of oxCNTs@30-QPEI, oxCNTs@50-QPEI, or oxCNTs@80-QPEI into 2 mL pure water. The suspensions were then ultra-sonicated for 5 min using a Hielscher UP200S high intensity ultrasonic processor at 40% amplitude and 0.5 cycles. Each sample was centrifuged at 1500 rpm for 15 min, and then the supernatant was diluted with pure water before measurement.

*ζ*-potential measurements were performed using a ZetaPlus -Brookhaven Instruments Corp. In a typical experiment, an aqueous 0.05 mg/mL dispersion of QPEI-functionalized oxCNTs was used, ten *ζ*-potential measurements were collected, and the results were averaged. Dynamic light scattering studies were carried out on an AXIOS-150/EX (Triton Hellas) system equipped with a 30 mW laser source, and an avalanche photodiode detector at 90° angle. In a typical experiment, an aqueous 0.05 mg/mL dispersion of QPEI-functionalized oxCNTs was used, at least ten measurements were collected, and the data were analyzed using the CONTIN algorithm to obtain the hydrodynamic radii distribution.

UV-vis spectra of the aqueous dispersions (1 mg/mL) were obtained by a Cary 100 Conc UV-visible spectrophotometer (Varian Inc., Mulgrave, Australia) in the range of 200–600 nm. Additionally, the colloid stability of the functionalized carbon nanotubes was evaluated at static conditions for 1, 6, and 12 months. Specifically, the dispersions obtained as described previously, were placed in vertically standing tubes and stored at room temperature. At each time point, 100 μL of the stock solutions from the very upper part was taken diluted in 1 mL water and the optical density (O.D.) of these dispersions was measured using UV-vis spectroscopy.

### 3.6. Escherichia coli Growth Inhibition Assay

The antibacterial activity of QPEI-functionalized oxCNTs was obtained by a bacteria growth inhibition assay. *Escherichia coli* XL1-blue bacteria expressing red fluorescent protein (RFP), from a plasmid-encoded gene, were grown in Luria–Bertani (LB) broth at 37 °C overnight, in a Stuart SI500 orbital shaker at approximately 200 rpm shaking speed in aerobic conditions. The culture was subsequently diluted to an optical density (O.D.) of 0.4 at 600 nm. The QPEI-functionalized oxCNTs were homogeneously dispersed in distilled water by sonication and added to the bacterial culture at concentrations ranging from 5 to 400 μg/mL. The assay was performed in a 96-well plate format in a 200 μL final volume. Fluorescence intensity over growth of untreated bacteria revealed that the optimum incubation time was 6 h, as the intensity reached a plateau (Figure S4). Thus, plates were incubated at 37 °C, shaking at 100 rpm in aerobic conditions for 6 h, and bacterial growth was monitored using the fluorescence intensity of red fluorescent protein, which was recorded at an emission wavelength of 590 nm by an Infinite M200 plate reader (Tecan group Ltd., Männedorf, Switzerland) at an excitation wavelength of 545 nm. In order to eliminate the effect of CNTs in the measured intensities, the initial values (at 0 h), although minor compared to those obtained after 6 h, were subtracted from the final measurements. For each treatment eight replicates were used and three independent experiments were performed. Untreated bacteria were used as control, representing 100% fluorescence intensity in Figure 8.

### 3.7. SEM Analysis of the Cellular Morphology

The morphology of the *Escherichia coli* bacteria after treatment with QPEI-functionalized oxCNTs was characterized by scanning electron microscopy (Jeol JSM 7401F Field Emission SEM). Specifically, cells were incubated with QPEI-functionalized oxCNTs at their 50% inhibitory concentration (IC-50), fixed with 3% glutaraldehyde in sodium cacodylate buffer (100 mM, pH = 7.1) for 6 h, transferred to a poly(l-lysine) coated glass cover slip, dehydrated using ethanol gradient (twice of 50%, 70%, 95%, and 100% ethanol for 10 min each), drying, and coated with gold in a sputter coater [74].

### 3.8. Synechococcus sp. PCC7942 Cyanobacteria Growth Inhibition Assay

*In vitro* anti-cyanobacterial activity of QPEI-functionalized oxCNTs was screened against *Synechococcus* sp. PCC 7942 bacteria. The unicellular cyanobacterium *Synechococcus* sp. PCC7942 was purchased from the Collection Nationale de Cultures de Microorganismes (CNCM), Institut Pasteur, Paris, France. The cyanobacterial cells were cultured in BG11 that additionally contained 20 mM HEPES-NaOH (pH = 7.5). The cultures were illuminated with white light from fluorescent lamps, providing a photosynthetic active radiation (PAR) of 100 μmol photons m^-2^ s^-1^, and were aerated with air containing 5% (*v*/*v*) CO_2_ in an orbital incubator (Galenkamp INR-400) at 31 °C [75]. QPEI-functionalized oxCNTs dispersed in distilled water using ultrasonication were added to the bacterial culture at concentrations ranged from 10 to 100 μg/mL. Cyanobacterial cell proliferation was monitored in terms of concentration of Chl *α*, determined in *N*,*N*-dimethylformamide (DMF) extracts [76]. To extract Chl *α*, the cell suspensions were centrifuged, DMF was added to the residue, and the resulting clear supernatant DMF extract was obtained after a second centrifugation.

Toxicity tests were performed in three replicate experiments using at least five geometrically scaled dilutions for each compound concentration. The cyanobacteria culture was inoculated in each test solution in the exponential growth phase at concentrations of approximately 1 μg Chl *α*/mL. The toxicity of the oxCNTs@QPEIs was evaluated as the effective concentrations (μg/mL) of the test substance inhibiting cell proliferation by 50% (IC-50) relative to the control cultures; in this test, the IC-50 values were calculated by the area under the growth curves (biomass) for each concentration of the hybrid materials, using non-linear regression of a 4-parameters logistic function. The related data are presented in Figure S6.

### 3.9. Measurements of Photosystem I and II Electron Transport Activities

Photo-induced electron transport rates were determined in *Synechococcus* permeaplasts [68] at room temperature oxymetrically (for each Photosystem) with a Clark-type oxygen electrode (DW1; Oxygraph, Hansatech, King’s Lynn, UK). *Synechococcus* sp. PCC 7942 bacteria were treated with lysozyme to obtain permeaplasts before the measurement of the photosynthetic electron transport activities [69]. The instrument was fitted with a slide projector to provide actinic illumination of samples. PSI activity was determined by measuring the rate of oxygen uptake, in the presence of the post-PSII electron transfer inhibitor 3-(3,4-dichlorophenyl)-1,10-dimethylurea (DCMU), using Na ascorbate/diaminodurene as an electron donor to PSI and methyl viologen as a post-PSI electron acceptor and mediator of oxygen uptake [77]. The reaction mixture (1 mL, in buffered BG11) contained permeaplasts (5 μg Chl *α*), diaminodurene (1 mM), Na-ascorbate (2 mM), methyl viologen (0.15 mM), and DCMU (0.01 mM). PSII activity was determined by measuring the rate of oxygen evolution, with water as electron donor and p-benzoquinone as post-PSII electron acceptor. The reaction mixture (1 mL in buffered BG11) contained permeaplasts (5 μg Chl *α*/mL) and p-benzoquinone (1 mM).

The redox state of P700 was determined *in vivo* using a PAM-101-modulated fluorometer (Heinz Walz GmbH, Effeltrich, Germany), equipped with an ED-800T emitter-detector, and PAM-102 units, following the procedure of Schreiber at al. [64]. The redox state of P700 was evaluated as the absorbance change around 820 nm (ΔA820/A820).

### 3.10. Cell Cytotoxicity

In this study, human prostate carcinoma cell line DU145 and 3T3 mouse fibroblasts, obtained from the American Type Culture Collection (ATCC, Manassas, VA, USA), were used. Cells were grown in low glucose supplemented D-MEM, containing 10% FBS, penicillin/streptomycin solution (100 U/mL + 100 μg/mL), and 2 mM L-Glutamine. Cells were incubated at 37 °C in a humidified atmosphere, containing 5% CO_2_ and sub-cultured, twice a week after detaching with a solution containing 0.05% (*w*/*v*) trypsin and 0.02% (*w*/*v*) EDTA. The cytotoxicity of oxCNTs and the QPEI functionalized oxCNTs was assessed employing MTT assay. DU145 cancer cells and 3T3 mouse fibroblasts were inoculated (10^4^ cells/well) into 96-well plates and incubated in complete media for 24 h. Cells were then treated with various concentrations of oxCNTs@QPEIs for 24 h. The mitochondrial redox function (translated as cell viability) of all cell groups was measured by the MTT assay. In brief, cell media was replaced with complete media containing MTT (10 μg/mL) and incubated at 37 °C in a 5% CO_2_ humidified atmosphere for 3 h. Then, the supernatant containing MTT was discarded and the produced formazan crystals were dissolved in isopropyl alcohol (100 μL per well) under shaking for 10 min at 100 rpm in a Stuart SI500 orbital shaker. Finally, the endpoint absorbance measurements at 540 nm were carried out, employing an Infinite M200 plate reader (Tecan group Ltd., Männedorf, Switzerland). Eight replicates were performed for each concentration, and the experiment was repeated in triplicate. The relative cell viability was calculated as cell survival percentage compared to cells that were treated only with complete media (control). Blank values measured in wells with isopropyl alcohol and no cells, were in all cases subtracted. 

## 4. Conclusions

In this study, negatively charged oxidized multi-walled carbon nanotubes (oxCNTs) were modified with positively charged quaternized hyperbranched poly(ethyleneimine) derivatives (QPEIs), through non-covalent functionalization. Specifically, three derivatives of hyperbranched poly(ethyleneimine), with a 30, 50, and 80% substitution degree of primary amino groups, were prepared and, subsequently, physically interacted with oxCNTs, yielding three novel QPEI functionalized oxCNTs, with QPEI loading ranged between 16–23%, approximately. Structural characterization of these hybrid materials using a variety of techniques, such as FTIR, RAMAN, SEM, TEM, AFM, etc., revealed the successful and homogenous anchoring of QPEIs on the oxCNTs surface. Furthermore, the microscopic techniques revealed the effective wrapping of the QPEI over the ox-CNTs. Contrary to previous studies on non-covalent functionalization of CNTs with PEI, the obtained hybrids efficiently dispersed in aqueous media, forming dispersions with excellent aqueous stability for over 12 months. To evaluate the antibacterial and anti-cyanobacterial properties of these hybrids, two types of gram(−) bacteria, an autotrophic (cyanobacterium *Synechococcus* sp. PCC 7942) and a heterotrophic (bacterium *Escherichia coli*), were used. It was found that all materials exhibited an enhanced, dose-dependent antibacterial and anti-cyanobacterial activity against both test organisms. The obtained IC-50 values were much lower compared to oxidized MWCNTs, revealing that the non-covalent attachment of QPEIs strongly induces the antibacterial/anti-cyanobacterial properties of the hybrid materials. These improved properties were attributed to the polycationic character of the oxCNTs@QPEIs, which enables the effective interaction of the hybrids with the bacteria membranes, facilitating their internalization into the cells. Moreover, the excellent aqueous dispersibility and stability of the hybrids, upon increasing the quaternization degree, further enhanced their activity. Indeed, the QPEI derivative containing the highest content of quaternary ammonium groups (80-QPEI) exhibited the highest performance, compared to the other QPEI derivatives. In the case of the photosynthetic bacteria, it was shown that the hybrid materials affect their photosynthetic apparatus by selective inhibition of the Photosystem (PS) I electron transport activity, while also reducing the photosynthetic electron transport in PSII. To the best of our knowledge, the QPEI functionalized hybrids are the first materials exhibiting strong anti-cyanobacterial properties, without the use of any antibiotic/herbicide. Furthermore, cytotoxicity studies on human prostate carcinoma DU145 cell line and the 3T3 mouse fibroblasts were performed, revealing that all hybrids exhibit high cytocompatibility in the concentration range in which they also exhibit high antibacterial and anti-cyanobacterial properties. These results suggest that QPEI-functionalized oxCNTs can be very attractive candidates as antibacterial and anti-cyanobacterial agents that can be used for potential applications in the disinfection industry, as well as for control of harmful cyanobacterial blooms.

## Figures and Tables

**Table 1:** IC-50 values of QPEI-functionalized oxCNTs on *Escherichia coli* XL1-blue bacteria.

Samples	IC-50 (μg/mL)
oxCNTs@30-QPEI	93.2
oxCNTs@50-QPEI	50.1
oxCNTs@80-QPEI	28.4

**Table 2:** IC-50 values of oxCNTs@QPEIs on cyanobacterium *Synechococcus* sp. PCC 7942.

Samples	IC-50 (μg/mL)
oxCNTs@30-QPEI	12.4
oxCNTs@50-QPEI	≤10
oxCNTs@80-QPEI	<10

## Supplementary Materials

The following are available online at https://www.mdpi.com/1424-8247/13/10/293/s1, Figure S1: FTIR spectra of oxCNTs, 30-QPEI, oxCNTs@30-QPEI, 50-QPEI and oxCNTs@50-QPEI, Figure S2: SEM images (upper part), AFM image and profile section (lower part) of oxCNTs, Figure S3: UV–vis absorption spectra of oxCNTs (a), oxCNTs@30-QPEI (b), oxCNTs@50-QPEI (c) and oxCNTs@80-QPEI (d) in aqueous solution (1 mg/mL), Figure S4: Fluorescence intensity change of RFP at 590 nm (excitation: 545 nm) upon *Escherichia coli* XL1-blue bacteria growth. Figure S5: Effect of oxCNTs on cell proliferation of cyanobacteria *Synechococcus* sp. PCC 7942 in the presence of different concentrations. Error bars represent mean ± SD for at least three independent experiments. Figure S6: Plot of the area under the growth curves of *Synechococcus* sp. PCC 7942 cells for each concentration of oxCNTs@PEIs versus the corresponding concentration as well as the relevant IC-50 calculations. Table S1: Elemental analysis results of ox-CNTs, QPEI and QPEI-functionalized ox-CNTs. Table S2: Photosystem II and I electron transport activities measured on *Synechococcus* sp. PCC 7942 permeaplasts in the presence of oxCNTs@80-QPEI. Table S3: Effects of oxCNTs@80-QPEI on the steady state oxidation of P700 (ΔA820/A820) by FR light in *Synechococcus* sp. PCC 7942 cells.
