# Supplementary material for: Cytotoxicity Effects of Water-Soluble Multi-Walled Carbon Nanotubes Decorated with Quaternized Hyperbranched Poly(ethyleneimine) Derivatives on Autotrophic and Heterotrophic Gram-Negative Bacteria
Source: Pharmaceuticals (Basel). 2020 Oct 6;13(10):293. doi: 10.3390/ph13100293 (PMC7601344; doi:10.3390/ph13100293)
Supplement: Supplementary file 1 [file pharmaceuticals-13-00293-s001.pdf]

Cytotoxicity Effects of Water-Soluble Multi-walled Carbon Nanotubes Decorated with Quaternized Hyperbranched Poly(ethyleneimine) Derivatives on Autotrophic and Heterotrophic Gram-negative Bacteria

Nikolaos S. Heliopoulos [1,2], Georgia Kythreoti [1,3], Kyriaki Marina Lyra [1], Katerina N. Panagiotaki [1], Aggeliki Papavasiliou [1], Elias Sakellis [1], Sergios Papageorgiou [1], Antonios Kouloumpis [4], Dimitrios Gournis [4], Fotios K. Katsaros [1], Kostas Stamatakis [3] and Zili Sideratou [1,*]

[1] Institute of Nanoscience and Nanotechnology, NCSR "Demokritos", 15310 Aghia Paraskevi, Greece; nikosheliopoulos@gmail.com (N.S.H); geokyth@bio.demokritos.gr (G.K.); kymarin@gmail.com (K.M.L.); knpanagiotaki@gmail.com (K.N.P.); a.papavasiliou@inn.demokritos.gr (A.P.); e.sakellis@inn.demokritos.gr (E.S.); s.papageorgiou@inn.demokritos.gr (S.P.); f.katsaros@inn.demokritos.gr (F.K.K.)

[2] Department of Industrial Design & Production Engineering, University of West Attica, 12244 Egaleo, Attiki, Greece

[3] Institute of Biosciences and Applications, NCSR "Demokritos", 15310 Aghia Paraskevi, Greece; kstam@bio.demokritos.gr

[4] Department of Material Science & Engineering, University of Ioannina, 45110 Ioannina, Greece; antoniokoul@gmail.com (A.K.); dgourni@uoi.gr (D.G)

* Correspondence: z.sideratou@inn.demokritos.gr; Tel.:+30-210-6503616

Supplementary Materials

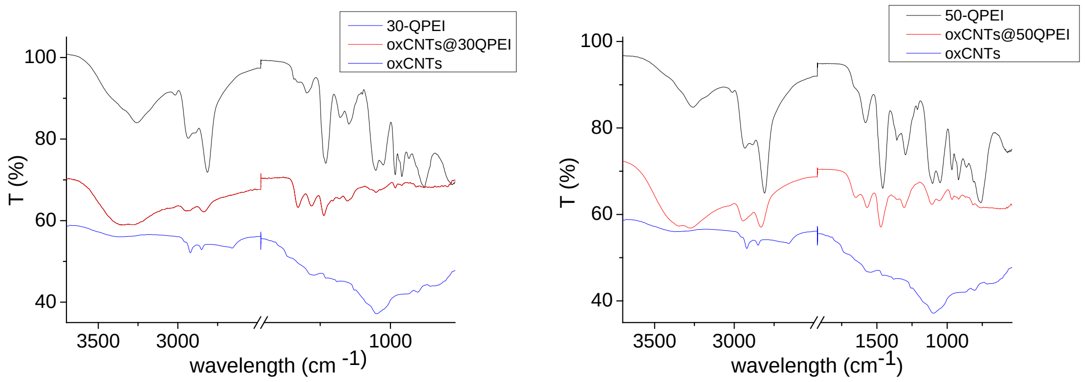

Figure S1. FTIR spectra of oxCNTs, 30-QPEI, oxCNTs@30-QPEI, 50-QPEI and oxCNTs@50-QPEI.

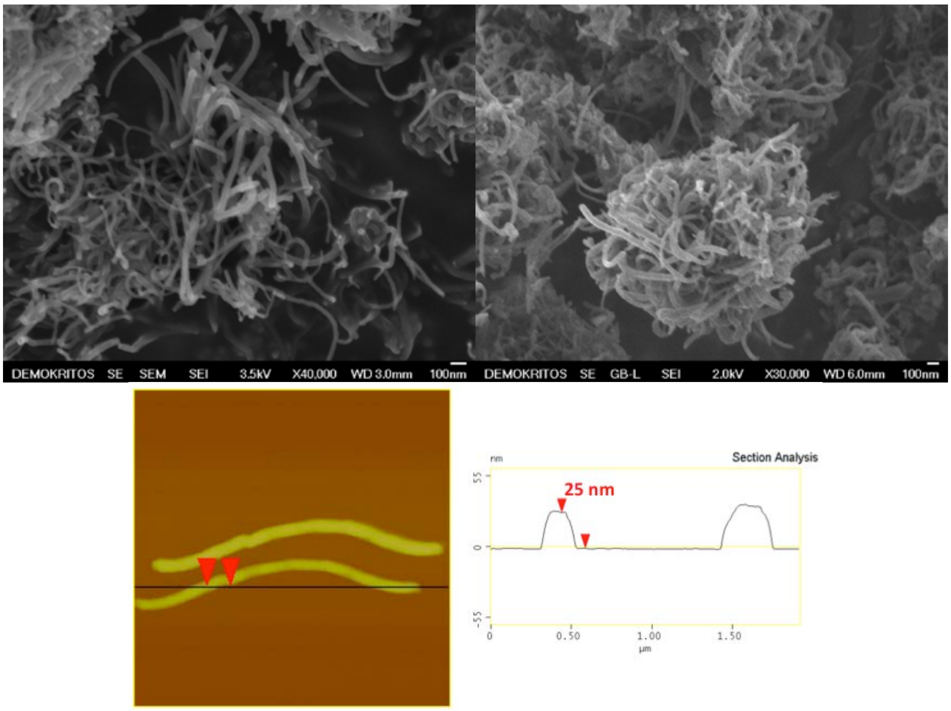

Figure S2. SEM images (upper part), AFM image and profile section (lower part) of oxCNTs.

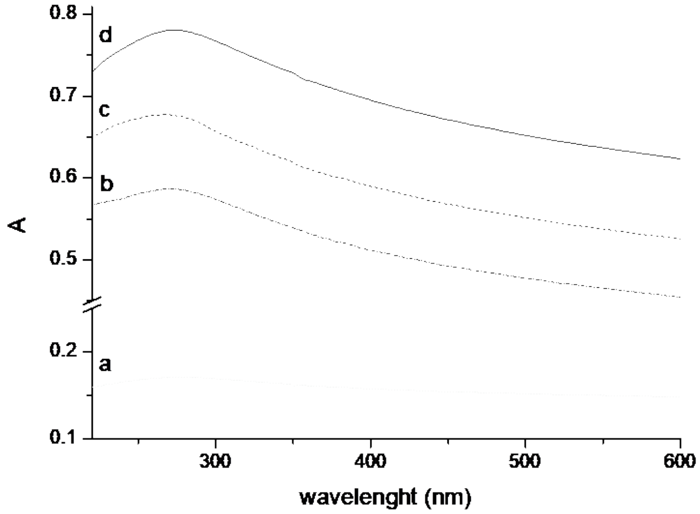

Figure S3. UV–vis absorption spectra of oxCNTs (a), oxCNTs@30-QPEI (b), oxCNTs@50-QPEI (c) and oxCNTs@80-QPEI (d) in aqueous solution (1 mg/mL).

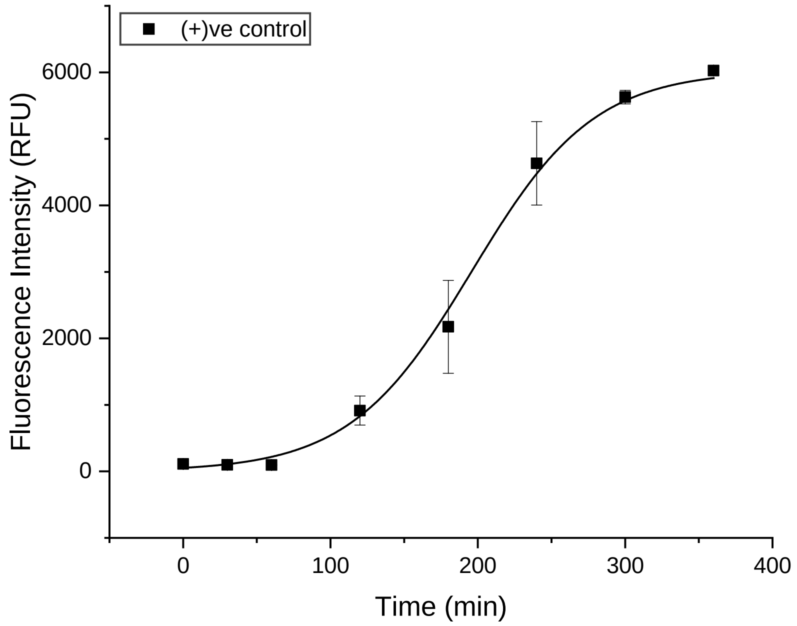

Figure S4. Fluorescence intensity change of RFP at 590 nm (excitation: 545 nm) upon *Escherichia* coli XL1-blue bacteria growth.

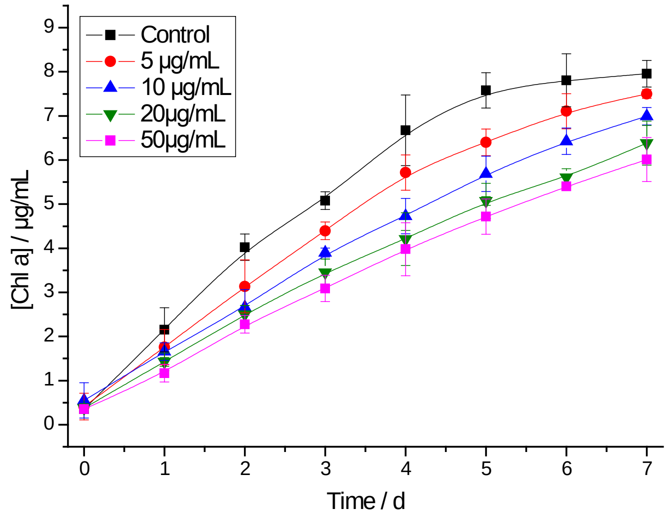

Figure S5. Effect of oxCNTs on cell proliferation of cyanobacteria Synechococcus sp. PCC 7942 in the presence of different concentrations. Error bars represent mean ± SD for at least three independent experiments.

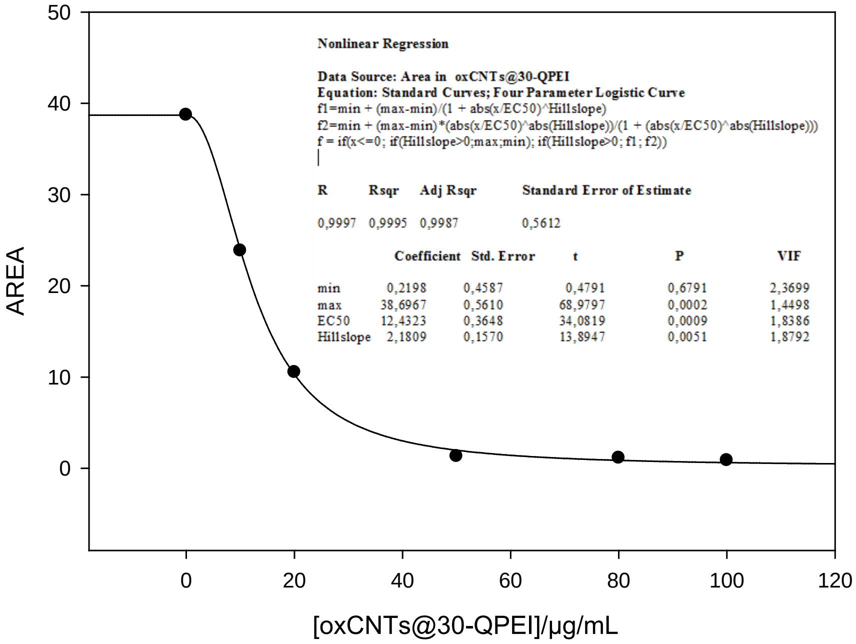
Nonlinear Regression
Data Source: Area in oxCNTs@30-QPEI
Equation: Standard Curves; Four Parameter Logistic Curve
f1=min + (max-min)/(1 + abs(x/EC50)^Hillslope)
f2=min + (max-min)*(abs(x/EC50)^abs(Hillslope))/(1 + (abs(x/EC50)^abs(Hillslope)))
f = if(x<=0; if(Hillslope>0;max;min); if(Hillslope>0; f1; f2))
R Rsqr Adj Rsqr Standard Error of Estimate
0,9997 0,9995 0,9987 0,5612
Coefficient Std. Error t P VIF
min 0,2198 0,4587 0,4791 0,6791 2,3699
max 38,6967 0,5610 68,9797 0,0002 1,4498
EC50 12,4323 0,3648 34,0819 0,0009 1,8386
Hillslope 2,1809 0,1570 13,8947 0,0051 1,8792
AREA
50
40
30
20
10
0
0
20
40
60
80
100
120
[oxCNTs@30-QPEI]/µg/mL

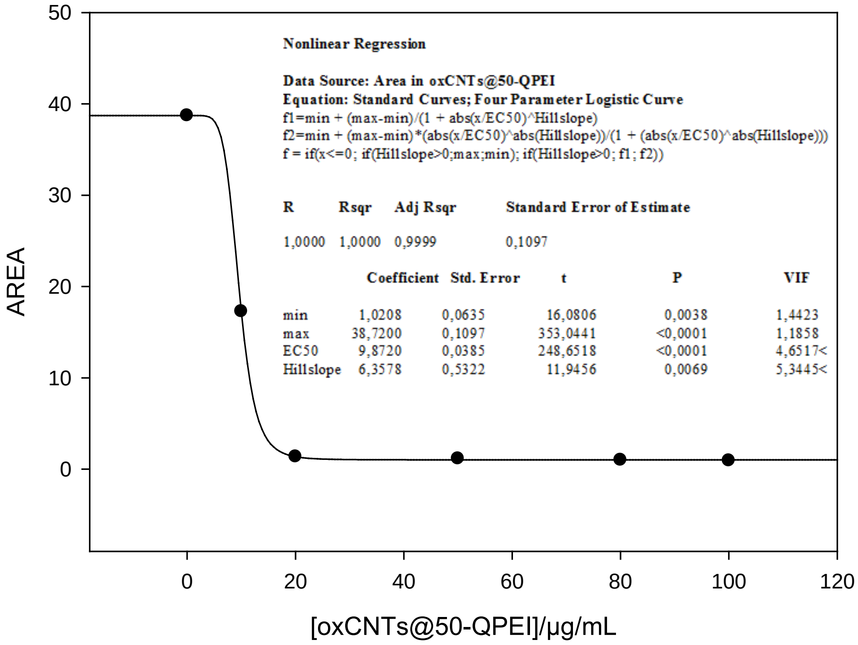
Nonlinear Regression
Data Source: Area in oxCNTs@50-QPEI
Equation: Standard Curves; Four Parameter Logistic Curve
f1=min + (max-min)/(1 + abs(x/EC50)^Hillslope)
f2=min + (max-min)*(abs(x/EC50)^abs(Hillslope))/(1 + (abs(x/EC50)^abs(Hillslope)))
f = if(x<=0; if(Hillslope>0;max;min); if(Hillslope>0; f1; f2))
R Rsqr Adj Rsqr Standard Error of Estimate
1,0000 1,0000 0,9999 0,1097
Coefficient Std. Error t P VIF
min 1,0208 0,0635 16,0806 0,0038 1,4423
max 38,7200 0,1097 353,0441 <0,0001 1,1858
EC50 9,8720 0,0385 248,6518 <0,0001 4,6517<
Hillslope 6,3578 0,5322 11,9456 0,0069 5,3445<
AREA
50
40
30
20
10
0
0
20
40
60
80
100
120
[oxCNTs@50-QPEI]/µg/mL

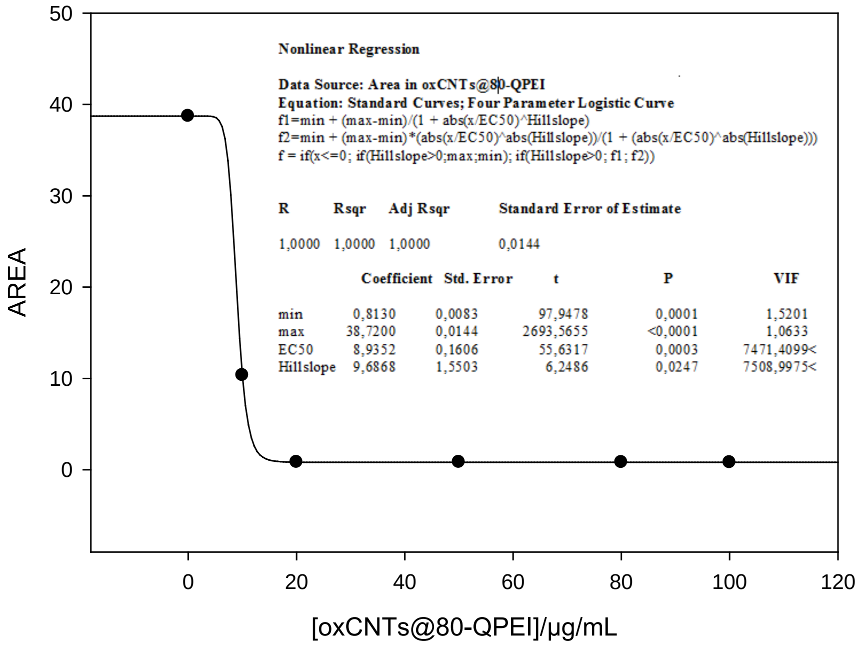

Figure S6. Plot of the area under the growth curves of Synechococcus sp. PCC 7942 cells for each concentration of oxCNTs@PEIs versus the corresponding concentration as well as the relevant IC-50 calculations. The IC-50 values were calculated as 12.4 μg/mL, 9.9 μg/mL and 8.9 μg/mL for oxCNTs@30-QPEI, oxCNTs@50-QPEI and oxCNTs@80-QPEI, respectively, using a non-linear regression of the 4-parameters logistic function.

Table S1. Elemental analysis results of ox-CNTs, QPEI and QPEI-functionalized ox-CNTs.

Sample elemental composition (wt%)				
**Sample**	**C**	**H**	**N**	**QPEI (% w/w)**
**ox-CNTs**	94.48	0.44	0.18	
**30-QPEI**	51.62	11.06	23.54	
**ox-CNTs@30-QPEI**	80.38	1.96	3.93	16.05 %
**50-QPEI**	51.49	11.87	24.48	
**ox-CNTs@50-QPEI**	81.67	2.99	5.02	19.92 %
**80-QPEI**	50.98	12.74	25.57	
**ox-CNTs@80-QPEI**	82.19	3.23	6.08	23.23 %

Table S2. Photosystem II and I electron transport activities measured on *Synechococcus* sp. PCC 7942 permeaplasts in the presence of oxCNTs@80-QPEI.

	**PSII activity (µmol O_2/mg Chl a * h)**	**PSI activity (µmol O_2/mg Chl a * h)**
**[oxCNTs] (µg/mL)**		
**0**	119.9	317.0
**20**	119.6	175.1
**25**	119.3	152.2
**100**	98.6	88.8
**200**	78.0	-
**250**	74.8	13.7
**[oxCNTs@80-QPEI] (µg/mL)**		
**0**	210.1	420.1
**20**	170.0	10.1
**25**	164.9	9.5
**80**	149.9	9.0
**200**	100.1	8.2
**250**	96.9	8.0

Table S3. Effects of oxCNTs@80-QPEI on the steady state oxidation of P700 (ΔA820/A820) by FR light in Synechococcus sp. PCC 7942 cells.

[oxCNTs@80-QPEI] (mg/mL)	PSI ($P700^+$) (ΔA820/A820) x (10^2)	PSI inhibition %
0	1.0502	-
10	0.6476	39
25	0.6387	40
50	0.3956	63
100	0.3003	72
150	0.2976	71
250	0.1951	82
